# An adult case of nephrotic syndrome presenting with pulmonary artery thrombosis: a case report

**DOI:** 10.1186/1752-1947-7-215

**Published:** 2013-08-23

**Authors:** Ikuyo Narita, Takeshi Fujita, Michiko Shimada, Reiichi Murakami, Yuko Shimaya, Norio Nakamura, Hideaki Yamabe, Ken Okumura

**Affiliations:** 1 Department of Cardiology, Respiratory Medicine and Nephrology, Hirosaki University Graduate School of Medicine, 5 Zaifu-cho, Hirosaki 036-8562, Japan; 2 Community Medicine, Hirosaki University Graduate School of Medicine, 5 Zaifu-cho, Hirosaki 036-8562, Japan

**Keywords:** Nephrotic syndrome, Thromboembolism

## Abstract

**Introduction:**

Pulmonary artery thrombosis is one of the most important complications in patients with nephrotic syndrome. It is well known among nephrologists, however, that this possibly lethal complication very rarely occurs before the diagnosis of nephrotic syndrome.

**Case presentation:**

A 21-year-old Japanese woman who had no specific medical history consulted a primary care clinic. Although she had been aware of the edema of her lower extremities for 2 weeks, her chief complaints were palpitations and chest pain, which had started the day before. An electrocardiogram and chest radiograph did not reveal any specific abnormalities. Because her etiology was not clear, she was referred to an emergency division in a hospital 2 days later. Although arterial blood gas analysis did not reveal hypoxemia, computed tomography revealed thrombi of the bilateral pulmonary arteries and left iliac vein. At this point, a laboratory examination confirmed the diagnosis of nephrotic syndrome. Subsequently, she was admitted, and anticoagulant therapy was initiated immediately. The next day, oral corticosteroid therapy was initiated, and an inferior vena cava filter was placed internally. Her proteinuria resolved after 3 weeks of treatment. The prompt and complete response to corticosteroid therapy suggested that minimal change disease was the etiology of the nephrotic syndrome and pulmonary artery thrombosis.

**Conclusions:**

An awareness regarding the complication of pulmonary artery thrombosis in nephrotic syndrome is important not only for nephrologists but for all clinicians. Contrast-enhanced computed tomography is crucial to detect pulmonary artery thrombosis.

## Introduction

Thromboembolism is one of the major complications of nephrotic syndrome (NS) [1]. In particular, pulmonary artery thrombosis is serious and often fatal [2]. It is well recognized among nephrologists, however, that this potentially lethal complication of NS very rarely occurs before the diagnosis of NS or that patients might not consult a nephrologist for their symptoms [3-6].

Here, we describe the case of a 21-year-old Japanese woman who had presented with pulmonary artery thrombosis, and was subsequently diagnosed with NS.

## Case presentation

A 21-year-old Japanese woman who had no specific medical history consulted a primary care clinic. Although she had been aware of the edema of her lower extremities for 2 weeks, her chief complaints were palpitations and chest pain, which had started the day before. An electrocardiogram (ECG) and chest radiograph did not reveal any specific abnormality. The next day she consulted another physician but her etiology was still not clear. She was referred to the emergency division in a municipal hospital 2 days later. Arterial blood gas analysis did not reveal hypoxemia. However, computed tomography (CT) revealed thrombi in her bilateral pulmonary arteries and left iliac vein, and a laboratory examination revealed NS. She was admitted and anticoagulant therapy was initiated immediately. The next day she was referred to a nephrologist at our university hospital.

**Figure 1:**
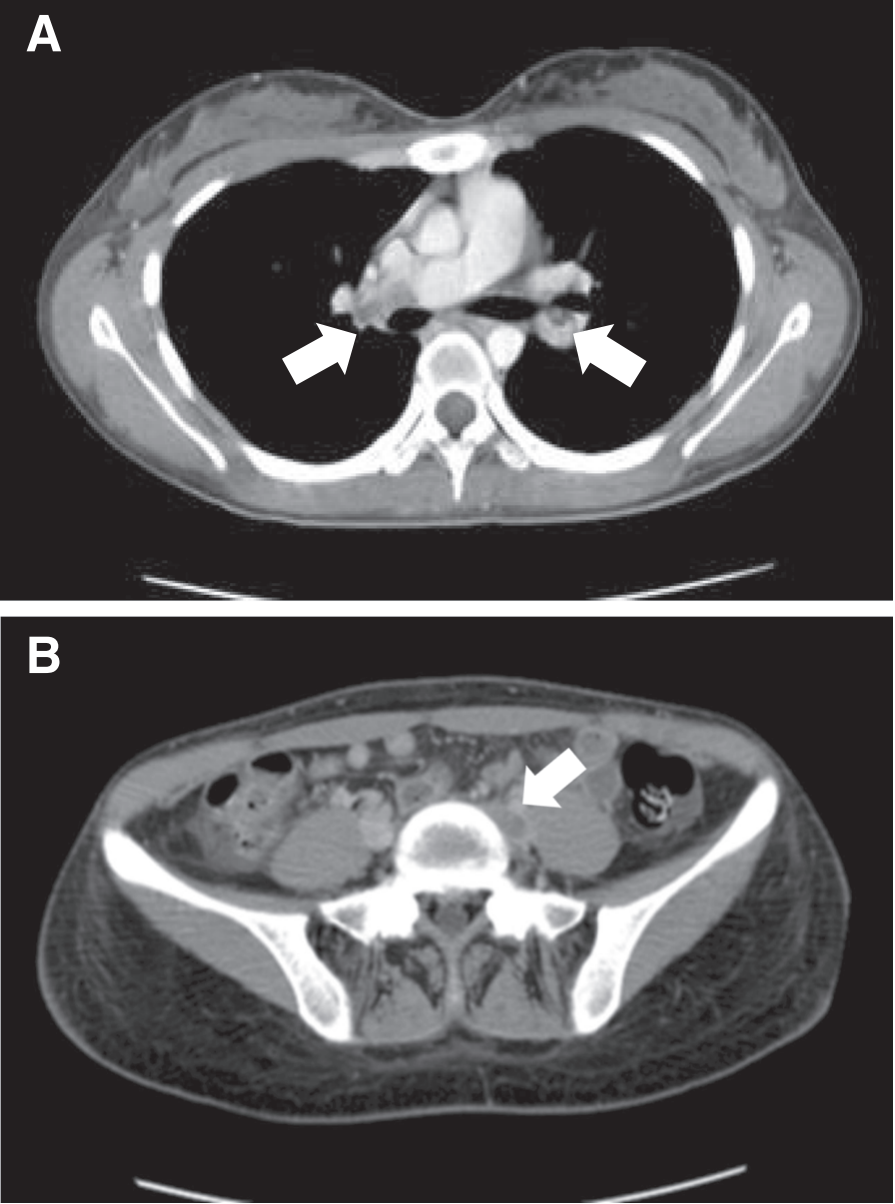
Computed tomography angiogram showing thrombi (arrows) in the pulmonary trunk, bilateral pulmonary artery (A) and left common iliac vein (B).

She had no family history of thromboembolism; she is a nonsmoker and not obese. She has never taken a contraceptive pill. On admission, her physical characteristics were: weight 53kg; height 166cm; body mass index 19.2kg/m^2^; blood pressure 110/62mmHg; pulse rate 120 beats/minute; respiratory rate 20 breaths/minute and saturation of peripheral oxygen (pulse oximetry) 97% (room air). Her physical examination was normal except for the edema of the lower extremities. The laboratory investigation data were as follows: leukocyte 9660 cells/μL; hemoglobin 14.8g/dL; platelets 329,000/μL; hematocrit 42.2%; blood urea nitrogen 19mg/dL; serum creatinine 1.26mg/dL; serum albumin 1.7g/dL; total protein 4.3g/dL; total cholesterol 497mg/dL; triglyceride 187mg/dL; the levels of serum complement C3 were 116mg/dL and C4 34mg/dL. Serum antinuclear antibody was negative. The coagulation profile revealed the following: prothrombin time 15.2 seconds; activated partial thromboplastin time (APTT) 84.5 seconds; D-dimer 6.9μg/mL (normal: 0 to 0.4μg/mL); fibrinogen 832mg/mL (normal: 150 to 410mg/mL); fibrin-fibrinogen degradation product 10.5μg/dL (normal: 0 to 5μg/dL); antithrombin III 70% (normal: 80% to 120%); protein C 29% (normal: 70% to 140%); and protein S 66% (normal: 65% to 135%). Urinary protein excretion was 14g/g creatinine, and microhematuria was absent. In addition, arterial blood gas analysis revealed the following: pH 7.46, carbon dioxide 35.5mmHg, partial pressure of oxygen 88.6mmHg, and bicarbonate 26.0mEq/L (room air). Her chest radiograph and ECG were normal. A CT angiogram revealed thrombi of her bilateral pulmonary arteries and left common iliac vein (Figure 1). Presumably, the pulmonary thrombosis originated from the thrombi of the left iliac vein.

**Figure 2:**
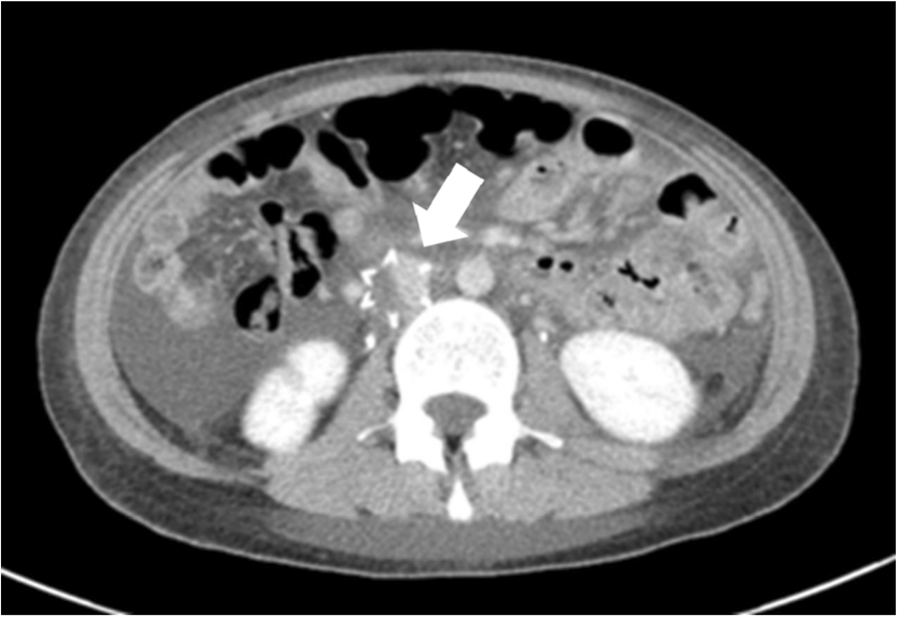
Computed tomography taken at day 14 showing capture of thrombi in the inferior vena cava filter (arrow).

Treatment of thrombosis was initiated with intravenous administration of heparin and urokinase. First, 18IU/kg of heparin per hour was initiated while adjusting the dose to maintain APTT between 60 and 85 seconds, thereafter, urokinase was infused at 10,000U/hour for 24 hours. Oral warfarin administration was also started, aiming at a prothrombin time-international normalized ratio of between 1.5 and 2.5, and heparin infusion was gradually decreased. An inferior vena cava (IVC) filter was placed percutaneously to avoid further development of pulmonary thrombosis by the left common iliac vein thrombi. Furthermore, corticosteroid therapy was initiated with prednisolone at a dose of 0.8mg/kg per day. Contrast-enhanced CT performed on day 14 revealed a reduction of the pulmonary thrombi and capture of the thrombi in the inferior vena cava (IVC) filter (Figure 2). The patient’s proteinuria resolved after 3 weeks of treatment. The prompt and complete response to the corticosteroid therapy suggested that minimal change disease was the etiology of NS and pulmonary artery thrombosis.

## Discussion

A higher risk of thromboembolism, both arterial and venous, has been reported in patients with NS [7]. It has also been reported that the magnitudes of proteinuria and hypoalbuminemia are related to venous thromboembolism, including pulmonary artery thrombosis, whereas a glomerular filtration rate test and multiple classic risk factors for atherosclerosis are related to the occurrence of the less frequent arterial thromboembolism [7].

The association between NS and thromboembolism was suggested in the literature as early as 1840 [8], however, the pathophysiology of thromboembolism in NS has not yet been clearly elucidated. Various hematological changes occur in NS and are related to incidences of thromboembolism [1,9]. In addition, the use of corticosteroids, immunosuppressants and diuretics have been suggested to be involved, whereas some studies have reported the antithrombotic effect of antiproteinuric therapies [10].

NS is associated with an increased protein synthesis rate in the liver, counterbalancing the urinary protein loss [1]. The shortage of several small molecular-size proteins remains because of the massive urinary loss. By contrast, the large molecular-size proteins escape the urinary loss and they are prominently increased because the increase in protein synthesis is generally nonselective.

Some of the important changes include decreased antithrombin III and protein C, which have anticoagulant effects, whereas some of the procoagulant factors such as fibrinogen, factor V and factor VIII are prominently increased [1,9]. Nevertheless, a clear molecular target for the prophylaxis has not yet been identified, and there is insufficient evidence to support the use of routine prophylactic anticoagulation therapy for all the patients with NS. The appropriate treatment and the prophylaxis for pulmonary artery thrombosis with NS are still under discussion because of the lack of large, prospective randomized trials.

Previous case reports of pulmonary artery thrombosis complicated with NS are very rare, particularly in adults. Presumably, this is largely because of the fact that thromboembolism is a common complication of NS for nephrologists. In the very rare cases that do occur, the patients initially complain of the symptoms derived from pulmonary artery thrombosis [2-4]. Palpitations or chest discomfort without obvious abnormality in ECG or chest radiograph particularly in young patients may not be regarded as serious. In fact, McPheeters *et al.* reported a case of 10-year-old boy with pulmonary artery thrombosis who died [6]. He had been treated for NS, and consulted an emergency room with a complaint of shortness of breath. After being stable on observation for several hours, he went home; however, he shortly returned to the hospital and died.

In our case, the patient was predisposed to a prothrombotic state because of severe hypoalbuminemia and hypercholesterolemia along with low levels of antithrombin III and protein C. She did not have any other prothrombotic risk factors, nor did she have symptoms which suggest collagen diseases such as systemic lupus erythematosus. She had been aware of the edema of the lower extremities for 2 weeks, however, her chief complaints on visiting the physician were palpitations and chest pain, which derived from pulmonary artery thrombosis. Although the first and the second physicians whom she consulted did recognize the possibility of NS, a CT was not performed until she visited a third physician in the emergency department 2 days later. Therefore the awareness of the complication of pulmonary artery embolism in NS is important not only for nephrologists but also for all other clinicians, particularly in primary care clinics or emergency rooms.

As previously reported [9,11,12], we successfully treated this case with urokinase, heparin, warfarin, and an IVC filter. The administration of urokinase in patients with stable hemodynamic variables is still controversial because of the risk of bleeding. Nevertheless, there was a significant risk of a recurrent thromboembolic event in this patient; in fact, the capture of the thrombus in the IVC filter indicated that the treatment was effective. Further investigation is necessary to clearly elucidate the appropriate treatment and prophylaxis for thromboembolism in patients with NS.

## Conclusions

In summary, we describe a case of a 21-year-old Japanese woman who presented with pulmonary artery thrombosis and was diagnosed with NS. The awareness of the complication of pulmonary artery embolism in NS is important not only for nephrologists but also for all other clinicians. Contrast-enhanced CT is essential for the diagnosis of pulmonary artery thrombosis.

## Consent

Written informed consent was obtained from the patient for publication of this case report and accompanying images. A copy of the written consent is available for review by the Editor-in-Chief of this journal.

## Competing interests

The authors declare that they have no competing interests.

## Authors’ contributions

IN and MS prepared the manuscript and performed the literature search. HY, NN and KO revised the manuscript. IN, RM, YS and TF treated the patient. All authors read and approved the final manuscript.
